# Extensive volatile loss during formation and differentiation of the Moon

**DOI:** 10.1038/ncomms8617

**Published:** 2015-07-03

**Authors:** Chizu Kato, Frederic Moynier, Maria C. Valdes, Jasmeet K. Dhaliwal, James M.D. Day

**Affiliations:** 1 Institut de Physique du Globe de Paris, Université Paris Diderot, CNRS UMR 7154, Paris 75005, France; 2 Department of Earth and Planetary Sciences and McDonnell Center for Space Sciences, Washington University in St Louis, St Louis, Missouri 63130, USA; 3 Institut Universitaire de France, Paris 75005, France; 4 Geosciences Research Division, Scripps Institution of Oceanography, La Jolla, California 92093-0244, USA

## Abstract

Low estimated lunar volatile contents, compared with Earth, are a fundamental observation for Earth–Moon system formation and lunar evolution. Here we present zinc isotope and abundance data for lunar crustal rocks to constrain the abundance of volatiles during the final stages of lunar differentiation. We find that ferroan anorthosites are isotopically heterogeneous, with some samples exhibiting high δ^66^Zn, along with alkali and magnesian suite samples. Since the plutonic samples were formed in the lunar crust, they were not subjected to degassing into vacuum. Instead, their compositions are consistent with enrichment of the silicate portions of the Moon in the heavier Zn isotopes. Because of the difference in δ^66^Zn between bulk silicate Earth and lunar basalts and crustal rocks, the volatile loss likely occurred in two stages: during the proto-lunar disk stage, where a fraction of lunar volatiles accreted onto Earth, and from degassing of a differentiating lunar magma ocean, implying the possibility of isolated, volatile-rich regions in the Moon's interior.

## Abstract (web summary)

Recent studies suggest that the Moon is not as volatile-poor as once thought, and that volatile elements should be concentrated in crustal materials. Here, the authors present Zn isotopic and abundance data as evidence of evaporative loss of volatiles during formation of the Moon, supporting alternative models.

## Graphical abstract

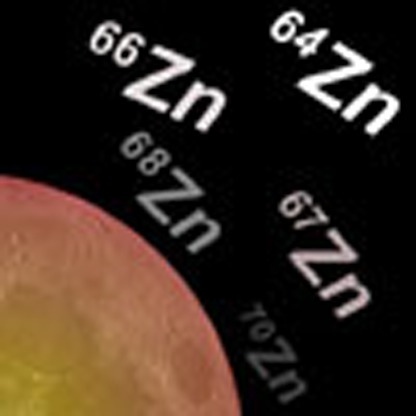

The present-day inventory of volatile elements in inner Solar System terrestrial bodies is a function of initial delivery and subsequent volatile depletion histories. Unravelling this history can, therefore, elucidate mechanisms of planetary growth and differentiation. In the case of the Earth–Moon system, the contrasting fate of volatiles, between a relatively volatile-poor Moon and a water-rich Earth, provides important evidence for their formation and evolution. Despite their clear importance, however, the fate of the volatile elements and the origin of the volatile depletion of the Moon is not well understood^1^,^2^. An important observation of Apollo lunar samples was their strong depletion in volatile elements compared with terrestrial samples^3^. More recent studies of the volatile content in the Moon have shown that isotopes of moderately volatile elements, such as Cl and Zn, are highly depleted, compared with bulk silicate Earth^4^,^5^,^6^. In addition, some lunar rocks are enriched in the heavier isotopes of Fe compared with bulk silicate Earth^7^. This observation was originally interpreted in terms of Fe volatilization during the giant impact^7^; however, igneous processes provide a more robust interpretation for these results^8^,^9^. In contrast, studies of OH contents and D/H in apatites from mare basalts, as well as Apollo pyroclastic glass beads, have argued for elevated volatile contents in portions of the lunar interior^10^,^11^,^12^,^13^. Reconciling these seemingly contrasting observations remains critical to examining models of lunar formation (for example, ref. 14) and of lunar mantle differentiation^15^, since a volatile-rich Moon is not a logical consequence of large-scale collisional events, or magma ocean differentiation^4^. To examine the origin of lunar volatiles, we employ high-precision Zn isotopes and abundances. Zinc is a moderately volatile element with a 50% condensation temperature of 726 K under solar nebula conditions^16^. Isotopic fractionation during magmatic processes has a negligible effect (<0.1‰ for δ^66^Zn; per mil deviation of ^66^Zn/^64^Zn from the JMC-Lyon standard)^17^. However, Zn isotopes are highly fractionated during high-temperature volatilization in planetary rocks and variations in δ^66^Zn larger than 1‰ in terrestrial igneous rocks have only been observed in association with evaporation events^18^,^19^. The absence of isotopic fractionation during igneous processes makes Zn isotopes powerful tracers of high-temperature evaporation during volatile depletion episodes in planets. Recent work on lunar samples has focused largely on using Zn isotopes to study mare basalts, regolith and pyroclastic glass beads^6^,^18^,^20^. These studies have shown that mare basalts are consistently enriched in the heavier Zn isotopes compared with terrestrial basalts, which has been interpreted as a consequence of a whole-scale evaporation event on the Moon^6^, or during magma ocean differentiation^4^. It has also been suggested that similar isotopic fractionation could possibly have occurred during eruptive degassing of volatiles from mare basalt lavas into vacuum^5^.

To obtain the most comprehensive understanding of the Zn isotopic composition of lunar samples, we investigated the Zn isotopic compositions and abundances of lunar crustal rocks, which include magnesian and alkali suite samples (collectively termed here as MGS) and ferroan anorthosite (FAN) plutonic rocks. MGS are interpreted to have formed within the FAN-dominated lunar crust^21^ and, therefore, have not experienced degassing in a vacuum. FAN samples were formed by crystallization of a plagioclase floatation crust above a magma ocean after >80% differentiation^21^. FANs have highly variable volatile element abundances, with Zn ranging from ∼0.1 to>50 p.p.m. (for example, ref. 22), possibly consistent with transport and redistribution of Zn at the lunar surface. New data are also presented for the pyroclastic green glass 15426 to further examine whether pyroclastic glasses are enriched in the light isotopes of Zn due to recondensation of isotopically light vapour^18^. Additional data are also reported for three lunar regolith samples and several mare basalt samples to confirm previously observed heavy Zn isotope enrichment in these lithologies^6^,^18^,^20^.

## Results

### Lunar basalts

**Figure 1:**
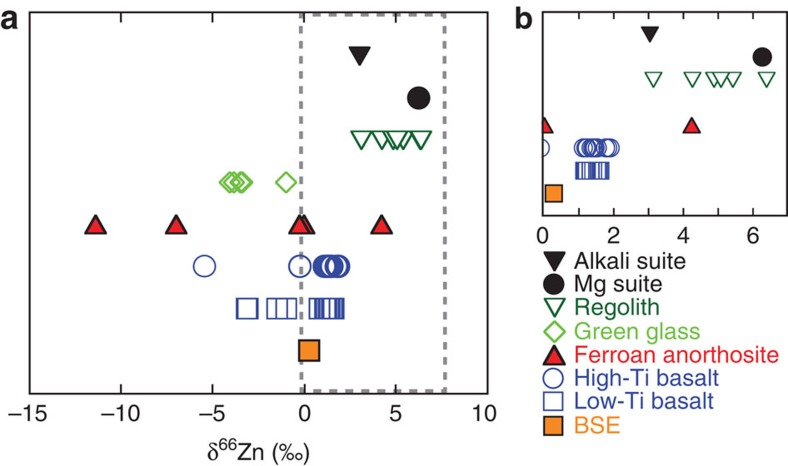
δ^66^Zn values for lunar rocks. The grey dashed box in **a** represents the magnified area shown in **b**. Lunar mare basalts, alkali and magnesium suite samples are enriched in the heavier isotopes of Zn in comparison with the terrestrial mantle composition (orange square). Ferroan anorthosites exhibit significant isotopic variability, to light and heavy Zn isotopic compositions. Isotopic data for lunar samples are from this study and refs 6, 18, 20. The bulk silicate Earth value is from ref. 17.

New data (Supplementary Table 1) for four high-Ti basalts (δ^66^Zn=+1.5±0.6‰, 2 s.d.) and three low-Ti basalts (δ^66^Zn=+1.5±0.4‰, 2 s.d.) (Supplementary Note 1) fall within the range observed previously for mare basalts^6^,^20^, and confirm that there is a consistent enrichment in the heavier isotopes of Zn compared with terrestrial basaltic rocks (δ^66^Zn=+0.28±0.05‰ for the bulk silicate Earth^17^) (Fig. 1). Combining our new data together with literature data^6^,^20^ (Supplementary Table 2), we calculate a new average value for lunar basalts of δ^66^Zn=+1.4±0.5‰ (2 s.d., *n*=26). A few exceptions (five samples out of 30) are isotopically lighter and are excluded from this average due to secondary condensation effects (Supplementary Discussion).

### Lunar crust and interior

**Figure 2:**
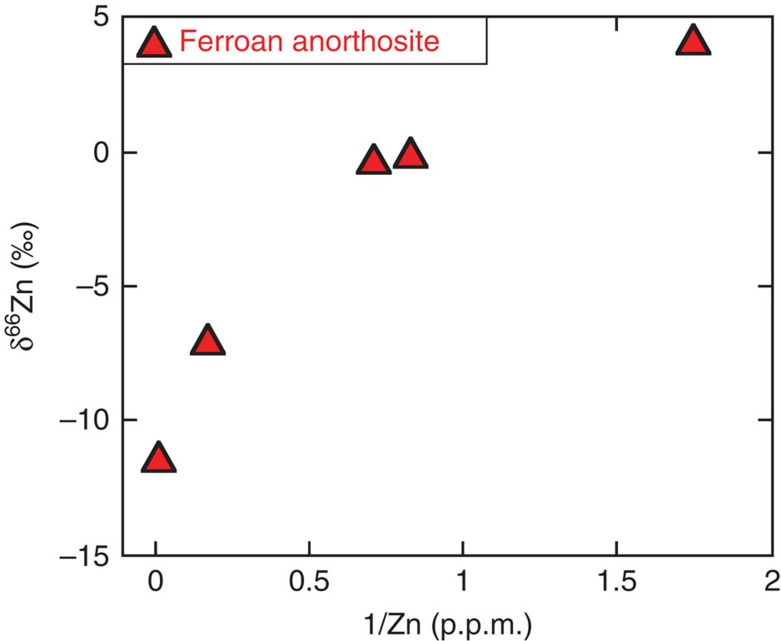
δ^66^Zn versus reciprocal Zn concentration in ferroan anorthosites. The correlation between δ^66^Zn and 1/Zn suggests redistribution and mixing of Zn between a surface reservoir rich in Zn that is isotopically light and a Zn poor, isotopically heavy primary magmatic reservoir.

In contrast to the relative homogeneity of mare basalts, FAN samples show large Zn isotopic variability (δ^66^Zn between −11.4 and +4.2‰) and the light isotope enrichment is correlated with the reciprocal Zn abundance (Fig. 2). In comparison, the two MGS rocks that we measured are both isotopically heavy (Supplementary Table 1).

### Pyroclastic glass and regolith

The pyroclastic green glass (15426) is enriched in Zn compared with mare basalts (∼50 p.p.m. versus ∼2 p.p.m., which is in good agreement with previous studies^22^) and is isotopically lighter than the mare basalts (δ^66^Zn=−0.98‰). This result confirms consistent light isotopic enrichment of Zn in both high- and low-Ti pyroclastic glass beads^18^,^20^, with δ^66^Zn for the pyroclastic glasses ranging from −1 to −4.1‰. Three regolith samples are enriched in the heavier isotopes of Zn, with δ^66^Zn up to +6.4‰, confirming previous results^18^,^20^ that the lunar regolith is isotopically heavier than lunar basalts through impact gardening processes (Supplementary Discussion). The Zn abundances of the different samples fall within the range of previous measurements: mare basalts: ∼1–5 p.p.m.; FAN: 0.6–75 p.p.m.; MGS: ∼3 p.p.m.; and pyroclastic green glass: ∼50 p.p.m. (Supplementary Table 2).

## Discussion

Enrichment in the lighter isotopes of Zn in Apollo 17 glasses was originally interpreted as the consequence of degassing of basaltic magma during a lava-fountaining event^18^. During magmatic ascent, the volatile elements are thought to have vapourized, leading to light isotope enrichment through kinetic isotopic fractionation. This vapour is then considered to have recondensed and coated the bead surfaces^18^. The new results on the Apollo 15 green glass (15426) offer the same interpretation for this pyroclastic deposit. The green glass 15426 is coarser (with a mean radius of the grains of ∼100 μm) than the two Apollo 17 glasses (mean radii of ∼50 μm)^23^. Therefore, a prediction, based on surface/volume, is for more limited light isotope enrichment of Apollo 15 glass beads due to surface/volume effects, as our results show (Apollo 15426 δ^66^Zn∼−1‰, [Zn]∼53 p.p.m. versus Apollo 74220 δ^66^Zn −3.4 to −4‰ and [Zn]∼230 p.p.m.).

Enrichment in the heavier isotopes of Zn in lunar mare basalts confirms that most lunar mare basalts have a homogeneous Zn isotopic composition, with 25 samples (out of 30) enriched by >1‰ (δ^66^Zn=+1.42±0.5‰; 2 s.d., *n*=26). It has previously been argued that the heavy isotope enrichment in mare basalts reflects a global phenomenon, where basalts representing four different Apollo mission sampling locations (Apollo 10, 12, 15 and 17), as well as unknown regions sampled by lunar meteorite LaPaz Icefield 02205, all show nearly identical enrichments^6^. The heavy Zn isotopic composition in the source of the mare basalts together, with the depletion in absolute abundances of moderately volatile elements, suggests that the lunar material suffered a whole-scale high-temperature evaporation process, with the preferential partitioning of the lighter isotopes of Zn in the vapour. This evaporation process most probably occurred during large-scale melting of the Moon, such as during the giant impact^6^.

Highland rocks represent the primary crust of the Moon and are the oldest known lunar samples (for example, ref. 24). The large range of Zn abundances in FAN samples suggests two possible scenarios: (1) the source of the FAN had a highly variable Zn content, which is reflected in the FAN samples, or (2) the Zn abundance represents secondary redistribution of Zn at the surface of the Moon during cataclasis of the FAN, as well as mixing with some chondritic components. Partial melting does not seem to extensively fractionate Fe/Zn due to the fact that these two elements share similar ionic radii^25^. The five different FAN samples analysed here have variable Fe/Zn (Supplementary Table 3), which, therefore, suggests that either the source of the anorthosites had variable Fe/Zn, that Fe and Zn were fractionated during plagioclase crystallization, or that the Zn elemental variations are a result of secondary evaporative processes. Since Zn isotopes are not fractionated by >0.2‰ during igneous processes^17^, the large Zn isotopic variations, which are correlated with the Zn abundance (Fig. 2), indicate that the Zn systematics of FAN are due to high-temperature evaporation effects. Thus, Zn-rich FAN contains a large fraction of an isotopically light vapour, while the most Zn-poor FAN (15415) retains primary magmatic characteristics.

Apollo sample 15415 is unbrecciated, pristine^26^ and is enriched in the heavier isotopes of Zn (δ^66^Zn=4.2‰). On the other hand, 65315, which is enriched in Zn (75 p.p.m.) is a brecciated cataclastic anorthosite with exogenously derived (meteoritic) highly siderophile element additions^27^. Similarly, 67955 is also a brecciated sample, formed from an impact melt, with a significant component of meteoritic siderophile elements^28^ and has disturbed Zn isotope systematics. These lines of evidence indicate that the brecciated samples incorporated isotopically light Zn-rich vapour during cataclasis or residence on the lunar surface. Transport of Zn at the surface of the Moon, during later impact bombardment of the crust and during the assimilation of exogenous Zn, implies that the Zn isotopic composition of cataclastic FAN samples do not represent the composition of their source reservoirs.

Significant enrichments in the heavy isotopes of Zn observed in the MGS, which solidified within the lunar crust, point towards a higher volatile depletion in their source than mare basalts. The origin of the MGS is generally interpreted as magmatic intrusions within the FAN crust (for example, ref. 21), formed by either a very high degree of partial melting^29^ or by assimilation of FAN crust^30^. Evaporation and condensation in a vacuum at the surface of the Moon has, therefore, played no role in the composition of these samples. Enriched heavier isotopes of Zn observed in the MGS points towards a source already depleted in volatile elements. In turn, this provides strong support to loss of Zn and moderately volatile elements during a whole-scale evaporation of the Moon early in its history either following the giant impact or during a lunar magma ocean phase^4^,^6^, and not locally during degassing, as has been proposed for some of the mare basalts (ref. 5). A third scenario would be that the impactor was already depleted in volatile elements before the giant impact (for example, refs 31, 32). The new data cannot constrain this scenario; however, a second process of volatile loss is required to explain the differences in Zn isotopic composition between mare basalts and pristine highland samples. A more likely scenario is, therefore, that volatile element of the Moon occurred during lunar formation and differentiation.

An outstanding question is how Zn and other moderately volatile elements were lost from the Moon, while water, which is far more volatile (for example, ref. 32), seems to have been retained in some samples (for example, refs 10, 11, 12, 13). It has been suggested that hydrodynamic escape after the giant impact was not a viable mechanism to explain the loss of water (or potassium) from the Earth–Moon system and that the volatile element content of the Moon was inherited from the precursor material^33^. However, this model fails to explain the extreme volatile depletion of the mare basalt source rocks, which are the most representative derivative rock type from the lunar mantle, or the later evolution of volatiles in the Moon revealed from crustal rocks. In a two-phase vapour-melt numerical model of the dynamical and thermodynamical evolution of the proto-lunar disk^34^, where the vapour phase is viscous due to magnetorotational instability, the volatile elements are predicted to accrete to Earth, leaving the material that formed the Moon enriched in refractory elements and correspondingly depleted in volatiles. Calculations of the chemical composition of the vapour of the lunar protoplanetary disk^35^ indicates that Zn enters the volatile phase of the disk at temperatures <3,000 K, leaving the Moon depleted in Zn and more volatile elements. Our new results support this mode of origin, but also suggest that continued volatile depletion in a lunar magma ocean is required^4^ to explain differences in δ^66^Zn between mare basalts and pristine lunar crustal rocks.

## Methods

### Sample preparation

One-gram chips of samples (Supplementary Note 1) were powdered with an agate mortar and pestle. Samples were then dissolved in a mixture of HF/HNO_3_ for several days in Teflon beakers at ∼140 °C. Zinc purification was achieved in a 0.25-ml column using AG-1X8 (200–400 mesh) anion-exchange resin. After applying the sample solution in HBr onto the resin, further HBr was added to remove elements causing matrix effects. Purified Zn was collected in a HNO_3_ solution following the procedure of ref. 18. The resulting Zn fraction was further purified in smaller 100-μl columns following the same ion-exchange chromatography procedure.

### Sample analysis

Samples were analysed using a Thermo-Fisher Neptune Plus multicollector inductively coupled plasma mass spectrometer at the Department of Earth and Planetary Sciences, Washington University in St Louis. Masses 62, 63, 64, 65, 66, 67 and 68 were measured by Faraday cups positioned, respectively, and the intensity of ^62^Ni was measured to control and correct the isobaric interference of ^64^Ni (ref. 6). All samples fell on the mass-dependent fractionation line (Supplementary Fig. 1), and the typical analytical uncertainty (2 s.d.), is 0.04‰ for δ^66^Zn and 0.05‰ for δ^67^Zn and δ^68^Zn. Instrumental mass bias was corrected by bracketing of the standard and the sample and is further discussed in ref. 17. The Zn concentrations were also obtained from multicollector inductively coupled plasma mass spectrometer data by comparing the intensity of the sample signal with that of the standard, with an estimated precision of ±10%.

## Additional information

**How to cite this article:** Kato, C. *et al.* Extensive volatile loss during formation and differentiation of the Moon. *Nat. Commun.* 6:7617 doi: 10.1038/ncomms8617 (2015).

## Supplementary Material

Supplementary Information Supplementary Figure 1, Supplementary Tables 1-3, Supplementary Note 1, Supplementary Discussion and Supplementary References
