# Supplementary material for: Extensive volatile loss during formation and differentiation of the Moon
Source: Nat Commun. 2015 Jul 3;6:7617. doi: 10.1038/ncomms8617 (PMC4506521; doi:10.1038/ncomms8617)
Supplement: Supplementary Information — Supplementary Figure 1, Supplementary Tables 1-3, Supplementary Note 1, Supplementary Discussion and Supplementary References [file ncomms8617-s1.pdf]

Supplementary Figure 1

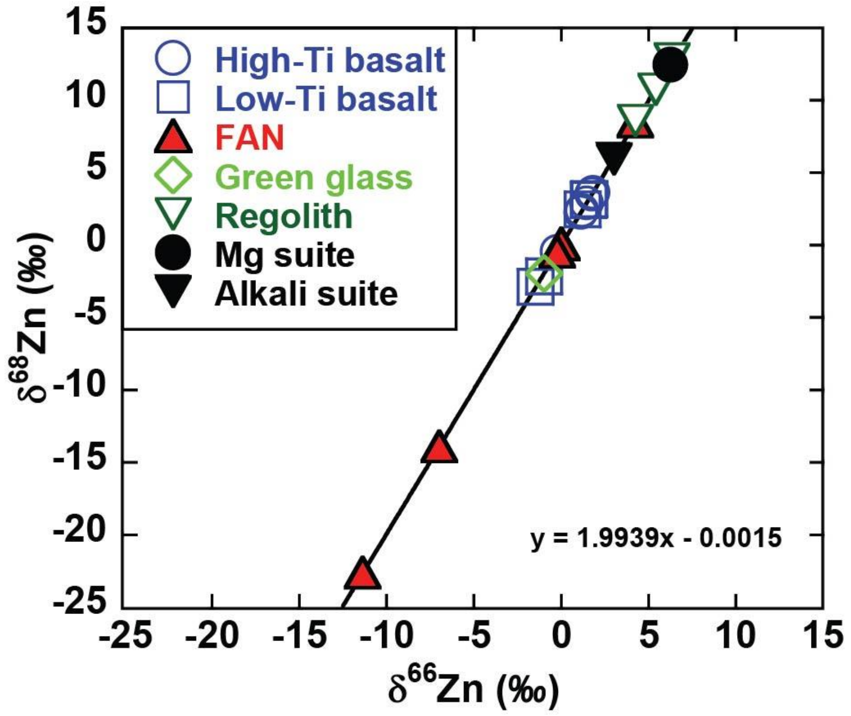

Supplementary Figure 1: δ^{68}Zn versus δ^{66}Zn for samples in this study. All samples fall on the mass dependent slope two line. FAN corresponds to Ferroan anorthosite.

Supplementary Table 1

Supplementary Table 1 Zn Isotopic values measured in this study

Sample	Type	$\delta^{66}Zn$	$\delta^{67}Zn$	$\delta^{68}Zn$	Zn (ppm)	Zn Lit. (ppm)
Low-Ti basalt						
**15499**	Basalt (Low-Ti)	1.23	1.88	2.47	0.9	n/d
**12012**	Olivine Basalt	1.62	2.39	3.17	1	n/d
**12040**	Olivine Basalt	1.58	2.39	3.12	0.8	0.78
**15016**	Vesicular Olivine Basalt	-1.47	-2.22	-2.88	2.1	1.8
**14053**	Mare Basalt (Al-rich)	-0.97	-1.5	-2.16	2.7	9.7
High-Ti basalt						
**70017**	Basalt (High-Ti)	1.20	1.79	2.39	2.7	3
**10003**	Basalt (High-Ti)	1.47	2.22	2.9	1.4	58.4
**70135**	Basalt (High-Ti)	1.13	1.73	2.25	1.8	39.6
**10057**	Ilmenite Basalt (High K)	1.83	2.73	3.62	5.4	20.5
**10057 (replicate)**	Ilmenite Basalt (High K)	1.77	2.63	3.5	5.4	20.5
**12005**	Ilmenite Basalt (High Mg/ Fe)	-0.22	-0.38	-0.46	0.8	n/d
Ferroan anorthosite						
**62255**	Anorthosite (w/melt)	0.00	0.15	0.09	1.2	0.31
**60015**	Cataclastic Anorthosite	-0.24	-0.39	-0.44	1.4	0.75
**65315**	Cataclastic Anorthosite	-11.37	-16.89	-22.62	75	73
**15415**	Ferroan Anorthosite	4.24	6.56	8.53	0.6	16.03
**67955**	Nortic Anorthosite	-6.98	-10.49	-13.89	5.9	6.73
Pyroclastic glass						
**15426**	Green Glass	-0.98	-1.44	-1.96	53	19
Lunar regolith						
**65701**	Soil	4.26	6.71	8.6	24.7	17.7
**78221**	Soil	5.42	8.06	10.76	31.6	25.6
**15041**	Trench Soil	6.35	10.01	12.86	17.3	14
Mg suite						
**72415**	Cataclastic Dunite	6.27	9.54	12.43	2.6	2.6
Alkali suite						
**77215**	Cataclastic Norite	3.04	4.53	5.95	3.6	2.98

Delta values are in parts per thousand (per mil). Zinc abundance literature values (Zn Lit.) are from the lunar compendium (http://curator.jsc.nasa.gov/lunar/lsc/).

Supplementary Table 2

Supplementary Table 2 Zn isotopic data and abundances from this study and published work

Sample	Type	$\delta^{66}Zn$	$\delta^{67}Zn$	$\delta^{68}Zn$	Zn (ppm)	Zn Lit. (ppm)
Low-Ti basalt						
**12002**[b]	Olivine Basalt	0.84	n/d	1.57	1.5	2.8
**12018**[b]	Olivine Basalt	-3.13	n/d	-6.37	1.3	2.3
**12018**[b]**(replicate)**	Olivine Basalt	-3.07	n/d	-5.76	1.3	2.3
**12021**[b]	Pigeonite Basalt	1.15	n/d	2.09	1.4	4.2
**12052**[b]	Pigeonite Basalt	1.34	n/d	2.76	1.1	9
**12065**[b]	Pigeonite Basalt	1.52	n/d	2.91	1.1	0.8
**12063**[b]	Ilmenite Basalt	1.18	n/d	2.49	1.0	3.4
**12016**[b]	Ilmenite Basalt	1.56	n/d	3.39	0.9	n/d
**15557**[b]	Ol-norm Basalt	1.34	n/d	2.85	0.6	1.3
**15555**[b]	Ol-norm Basalt	1.56	n/d	2.98	1.1	1
**15058**[b]	Qtz-norm Basalt	1.41	n/d	2.76	1.7	0.9
**15065**[b]	Qtz-norm Basalt	1.33	n/d	2.87	1.1	1.3
**LAP 02205**[b]	Meteorite-Olivine Basalt	1.20	n/d	2.32	n/d	2.9
**15499**[a]	Basalt (Low-Ti)	1.23	1.88	2.47	0.9	n/d
**12012**[a]	Olivine Basalt	1.62	2.39	3.17	1.0	n/d
**12040**[a]	Olivine Basalt	1.58	2.39	3.12	0.8	0.78
**15016**[a]	Vesicular Olivine Basalt	-1.47	-2.22	-2.88	2.1	1.8
**14053**[a]	Mare Basalt (Al-rich)	-0.97	-1.5	-2.16	2.7	9.7
High-Ti basalt						
**10017, 52**[c]	Ilmenite Basalt (High K)	-5.42	-8.02	-10.63	12.1	48
**10022, 47**[c]	Ilmenite Basalt (High K)	1.24	2.31	2.97	2.2	2.9
**10024, 108**[c]	Ilmenite Basalt (High K)	1.40	2.15	2.86	2.8	14
**70215, 323**[c]	Ilmenite Basalt	1.44	2.59	2.55	0.8	4
**71055, 234**[c]	Ilmenite Basalt	1.35	1.94	2.74	2.1	2.5
**74255, 191**[c]	Ilmenite Basalt	1.77	2.68	3.07	1.2	5.4

**75055, 118[c]**	Ilmenite Basalt	1.53	2.34	2.85	1.3	7
**75075, 193[c]**	Ilmenite Basalt	1.90	4.78	3.06	2.0	5
**70017[a]**	Basalt (High-Ti)	1.20	1.79	2.39	2.7	3
**10003[a]**	Basalt (High-Ti)	1.47	2.22	2.9	1.4	58.4
**70135[a]**	Basalt (High-Ti)	1.13	1.73	2.25	1.8	39.6
**10057[a]**	Ilmenite Basalt (High K)	1.83	2.73	3.62	5.4	20.5
**10057 (replicate)[a]**	Ilmenite Basalt (High K)	1.77	2.63	3.50	5.4	20.5
**12005[a]**	Ilmenite Basalt (High Mg/ Fe)	-0.22	-0.38	-0.46	0.8	n/d
Ferroan anorthosite						
**62255[a]**	Anorthosite (w/ melt)	0.00	0.15	0.09	1.2	0.31
**60015[a]**	Cataclastic Anorthosite	-0.24	-0.39	-0.44	1.4	0.75
**65315[a]**	Cataclastic Anorthosite	-11.37	-16.89	-22.62	75	73
**15415[a]**	Ferroan Anorthosite	4.24	6.56	8.53	0.6	16.03
**67955[a]**	Nortic Anorthosite	-6.98	-10.49	-13.89	5.9	6.73
Pyroclastic glass						
**74220, 793[d]**	Soil (clod)	-3.47	-5.21	-6.87	231	248
**74220, 849[d]**	Soil (clod)	-3.83	-5.72	-7.4	140	248

**74420, 860[c]**	Soil (clod)	-3.37	-5.06	-6.78	161	248
**74001, 2207[c]**	Soil (clod)	-4.05	3.66	-7.96	129	156
**15426[a]**	Green Glass	-0.98	-1.44	-1.96	53	19
Lunar regolith						
**65701[a]**	Soil	4.26	6.71	8.60	24.7	17.7
**78221[a]**	Soil	5.42	8.06	10.76	31.6	25.6
**15041[a]**	Trench Soil	6.35	10.01	12.86	17.3	14
**15021, 169[c]**	Soil	4.88	6.96	9.52	21.0	13.9
**15231, 200[d]**	Soil	6.39	9.52	12.69	n/d	16.5
**70181, 170[c]**	Soil	3.14	4.79	6.25	32.9	13 (47)
**79221, 143[c]**	Soil	5.08	7.63	10.05	31.8	21
Mg suite						
**72415[a]**	Cataclastic Dunite	6.27	9.54	12.43	2.6	2.6
Alkali suite						
**77215[a]**	Cataclastic Norite	3.04	4.53	5.95	3.6	2.98

Delta values are in parts per thousand (per mil). Zinc isotopic data are from the following: a = this study; b = ref. 1; c = ref. 2; d = ref. 3 Zn abundance literature values are from the lunar compendium.

Supplementary Table 3

Supplementary Table 3 Ratios of Fe/Zn in lunar highland (FAN, Mg suite, alkali suite) samples

Sample	Type	Fe (wt.%)	Zn (ppm)	Fe/Zn
Ferroan anorthosite				
**62255**	Anorthosite (with melt)	0.16	1.2	1288
**60015**	Cataclastic Anorthosite	0.19	1.4	1349
**65315**	Cataclastic Anorthosite	0.24	75	32
**15415**	Ferroan Anorthosite	0.16	0.6	2803
**67955**	Nortic Anorthosite	3.21	5.9	5447
Mg Suite				
**72415**	Cataclastic Dunite	8.81	2.6	33868
Alkali Suite				
**77215**	Cataclastic Norite	7.83	3.6	21799

Zinc concentration values were measured in this study, iron wt.% values are from the lunar compendium.

Supplementary Note 1

We analysed the Zn isotopic composition of 21 Apollo lunar samples:

- Five low-Ti basalts (olivine basalts 12012 and 12040, vesicular olivine basalt 15016, basalt 15499, aluminous mare basalt 14053).
- Five high-Ti basalts (basalts 10003, 70017, and 70135 and ilmenite basalts 10057 and 12005).
- Five highland anorthosites (anorthosite 65315, cataclastic anorthosites 62255 and 60015, the very primitive unbrecciated ferroan anorthosite 15415, noritic anorthosite 67955).
- Three regolith samples (65701, 78221 and trench soil 15041).
- One cataclastic dunite, 72415, which is a highland sample from the magnesian suite.
- One cataclastic norite, 77215, which is a highland sample from the alkali suite
- One pyroclastic green glass, 15426.

Samples were obtained from the Curation and Analysis Planning Team for Extraterrestrial Materials (CAPTEM) and the Apollo samples are curated in dedicated facilities at the NASA Johnson Space Center.

Supplementary discussion

Five basalt samples (out of 30) are isotopically light (δ^{66}Zn = -5.42 to -0.22‰) compared with the other mare basalts and terrestrial basaltic rocks. They are also enriched in Zn compared to the average of lunar basalts (2.2 ± 4.5 ppm, 2 sd). 10017 (δ^{66}Zn = -5.42‰), the basalt with the lightest Zn isotope composition, has been contaminated by an isotopically light source of Zn during its formation. This either occurred due to vapour degassed from the lava itself, or from material transported by the gardening of the lunar surface since the emplacement and crystallization of the basalt. The long cosmic ray exposure age of 10017 (~440-510 Ma; [4, 5]), and exposure ages of other samples (typically <400Ma) are certainly permissive with condensation-deposition on the outsides of samples. Thus, the isotopic composition of sample 10017, as well as other isotopically light samples, is not representative of source composition. These observations are supported by the evidence for correlated Zn abundances and cataclasis in FAN samples. Therefore, we exclude these isotopically light values of mare basalts from the discussion of the bulk Moon composition.

Regolith samples are all enriched in the heavier isotopes of Zn compared with mare basalts, with $\delta^{66}Zn$ ranging from +4.26 to +6.35‰ as previously observed[2, 3]. The regolith is known to be enriched in heavier isotopes of other moderately volatile elements compared with the mare basalts (e.g., Sulfur[6, 7], Potassium, $\delta^{41}K$ = +5 to +12.7‰[8, 9]), due to sputtering effects of solar wind and micrometeorite impacts, with the lighter isotopes selectively vaporized, with the vapor either escaping the Moon, or being sampled on the outsides of samples with long-exposure ages (c.f., 10017).

Supplementary References

1. Paniello, R. C., Day, J. M. D. & Moynier, F. Zinc isotopic evidence for the origin of the Moon. *Nature* **490,** 376–379 (2012).
2. Herzog, G. F., Moynier, F., Albarède, F. & Berezhnoy, A.A. Isotopic and elemental abundances of copper and zinc in lunar samples, Zagami, Pele's hairs, and a terrestrial basalt. *Geochim. Cosmochim. Acta.* **73,** 5884-5904 (2009).
3. Moynier, F., Albarède, F. & Herzog, G.F. Isotopic composition of zinc, copper, and iron in lunar samples. *Geochim. Cosmochim. Acta.* **70,** 6103–6117 (2006).
4. Turner, G. Argon-40/argon-39 dating of lunar rock samples. *Proc. Apollo 11 Lunar Sci. Conf.* 1665-1684 (1970).
5. Marti, K., Lugmair, G.W. & Urey, H.C. Solar wind gases, cosmic ray spallation products and the irradiation history of Apollo 11 samples. *Proc. Apollo 11 Lunar Sci. Conf.* 1357-1367 (1970).
6. Clayton, R. N., Mayeda, T. K. & Hurd, J. M. Loss of oxygen, silicon, sulfur, and potassium from the lunar regolith. *Proc. Lunar Conf.* **5,** 1801-1809 (1974).
7. Thode, H. G. & Rees, C. E. Measurement of sulphur concentrations and the isotope ratios

$^{33}S/^{32}S$, $^{34}S/^{32}S$, and $^{36}S/^{32}S$ in Apollo 12 samples. *Earth Planet. Sci. Lett.* **12,** 434-438 (1971).

8. Humayun, M. & Clayton, R. N. Precise determination of the isotopic composition of potassium: application to terrestrial rocks and lunar soils. *Geochim. Cosmochim. Acta*. **59,** 2115-2130 (1995a).

9. Humayun, M. &, Clayton, R. N. Potassium isotope geochemistry: genetic implications of volatile element depletion. *Geochim. Cosmochim. Acta.* **59,** 2131-2148 (1995b).
